# 

**Published:** 1905-10

**Authors:** 


October, 1905. Vol. XXVI. No. 10.

### THE INTERNATIONAL DENTAL JOURNAL.

JAMES TRUMAN, D.D.S., Editor,
PHILADELPHIA.

Collaborator: LAWRENCE W. BAKER, D.M.D.

BOSTON.

LIBRARY
SURGEON GENERAL'S OFFICE
OCT. 5-1905

LIBRARY
INDEXED
S. G. O.

### Summary of Contents.

(*For details, see p. 2.*)

ORIGINAL COMMUNICATIONS.

REVIEWS OF DENTAL LITERATURE.

REPORTS OF SOCIETY MEETINGS.

EDITORIAL.

MISCELLANY.

CURRENT NEWS.

*$2.50 a Year, in Advance. Single Copies, 25 Cents.*

PUBLISHED MONTHLY BY

The International Dental Publication Company,

227-231 SOUTH SIXTH ST., PHILADELPHIA.

EDITORIAL OFFICE, 4505 CHESTER AVENUE, PHILADELPHIA, PA.

Printed by J. B. Lippincott Company, Philadelphia.

Entered at the Post-Office at Philadelphia, and admitted for transmission through the mails at second-class rates.

